# Economic and health impacts of the Change4Life Food Scanner app: Findings from a randomized pilot and feasibility study

**DOI:** 10.3389/fnut.2023.1125542

**Published:** 2023-03-16

**Authors:** Sundus Mahdi, Nicola J. Buckland, Jim Chilcott

**Affiliations:** ^1^School of Health and Related Research, University of Sheffield, Sheffield, United Kingdom; ^2^Department of Psychology, University of Sheffield, Sheffield, United Kingdom

**Keywords:** economic evaluation, mobile applications, childhood obesity prevention, diet and nutrition, mHealth, digital interventions, feasibility study

## Abstract

**Introduction:**

The UK Government developed the Change4Life Food Scanner app to provide families with engaging feedback on the nutritional content of packaged foods. There is a lack of research exploring the cost-effectiveness of dietary health promotion apps.

**Methods:**

Through stakeholder engagement, a conceptual model was developed, outlining the pathway by which the Food Scanner app leads to proximal and distal outcomes. The conceptual model informed the development of a pilot randomized controlled trial which investigated the feasibility and acceptability of evaluating clinical outcomes in children and economic effectiveness of the Food Scanner app through a cost-consequence analysis. Parents of 4–11 years-olds (*n* = 126) were randomized into an app exposure condition (*n* = 62), or no intervention control (*n* = 64). Parent-reported Child Health Utility 9 Dimension (CHU9D) outcomes were collected alongside child healthcare resource use and associated costs, school absenteeism and parent productivity losses at baseline and 3 months follow up. Results for the CHU9D were converted into utility scores based on UK adult preference weights. Sensitivity analysis accounted for outliers and multiple imputation methods were adopted for the handling of missing data.

**Results:**

64 participants (51%) completed the study (intervention: *n* = 29; control: *n* = 35). There was a mean reduction in quality adjusted life years between groups over the trial period of –0.004 (SD = 0.024, 95% CI: –0.005; 0.012). There was a mean reduction in healthcare costs of –£30.77 (SD = 230.97; 95% CI: –£113.80; £52.26) and a mean reduction in workplace productivity losses of –£64.24 (SD = 241.66, 95% CI: –£147.54; £19.07) within the intervention arm, compared to the control arm, over the data collection period. Similar findings were apparent after multiple imputation.

**Discussion:**

Modest mean differences between study arms may have been due to the exploration of distal outcomes over a short follow-up period. The study was also disrupted due to the coronavirus pandemic, which may have confounded healthcare resource data. Although measures adopted were deemed feasible, the study highlighted difficulties in obtaining data on app development and maintenance costs, as well as the importance of economic modeling to predict long-term outcomes that may not be reliably captured over the short-term.

**Clinical trial registration:**

https://osf.io/, identifier 62hzt.

## 1. Introduction

Childhood overweight and obesity is a growing public health problem. In the UK alone, approximately 23% of 4–5 years old children and 38% of 10–11 years old children are impacted (1). Childhood obesity increases the risk of non-communicable diseases, such as asthma, sleep apnea, musculoskeletal problems, and psychological problems (2). This creates a greater demand for healthcare resource use, therefore negatively impacting on limited healthcare budgets. Direct medical costs of obesity are estimated at £6.1 billion to the UK National Health Service (NHS) (3), and $14 billion in the United States (4, 5). The rising trends in overweight and obesity has been associated with the growing availability of high density and nutritionally poor foods (6).

The use of smartphones has grown extensively. Recent figures suggest that 88% of the UK online adult population engage with mobile applications (7), whilst over half of US smartphone users have used a health app (8). Mobile apps have demonstrable beneficial impacts on weight reduction and dietary choices (9), whilst offering flexibility in their administration and use. They have the potential to reach diverse populations at low cost and may be provided by public health agencies as a public good. As such, there has been a growing number of dietary interventions delivered *via* smartphone apps (10, 11). Despite being deemed a cost-effective method to deliver dietary interventions (12), few studies have considered economic and cost outcomes within their analyses, with little guidance available to aid this process. As such, it has been flagged that further research is needed on how best to integrate economic factors into intervention design (13).

Unlike conventional healthcare interventions (e.g., pharmaceutical), mobile apps have their own methodological issues within evaluations, therefore require specific guidance to aid cost-effectiveness analyses (13–15). Current recommendations for practice have included implications for resource use and benefit measurement pertaining to app evolvement (15), including development, implementation, and updates up to eventual obsolescence (14); intervention costs based on study sample size or potential population reach (15); extended health benefits such as spill-over effects of the intervention onto social networks (15); and non-health care impacts such as productivity (15). Given this, cost per quality adjusted life years (QALY) within economic analysis have been deemed unlikely to capture health and non-health impacts of mobile health (mHealth) interventions. Instead, cost-consequence analysis, where a clear breakdown of costs and various benefits, has been recommended (15). This allows decision makers to use only the relevant aspects of this breakdown for their own local contexts.

Economic evaluations of dietary app-based interventions are only just emerging. The SWAP-IT trial aimed to reduce energy-dense foods packed in lunchboxes. The intervention included an mHealth component which provided support on healthy lunchbox preparation to parents of primary school children in Australia (16). The intervention adopted the use of an existing school app to communicate health promotion messages *via* push-notifications to support packing of healthy lunchboxes. Non-app components included the dissemination of resources to parents alongside lunchbox nutrition guidelines. Within a trial-based economic evaluation, costs relating to the mHealth component only included graphic design revisions and liaison time. Overall the intervention was deemed cost-effective at reducing energy from energy-dense, poor nutrient foods (17). Similarly, LifeLab Plus targets improvements in dietary behaviors in adolescents in the UK. The multicomponent intervention included education modules, training for teachers, and an interactive mobile app component with gaming features. A Markov model was developed to estimate the costs, benefits and cost-effectiveness of the intervention in comparison to usual schooling (18). The model assumed that intervention effects were sustained for 4 years, and then diminished to no effect over 10 years. The European Quality of Life 5 Dimensions 3 Level (EQ-5D-3L) was used to estimate quality of life outcomes. App costs were incorporated as capital costs and assumed to last 10 years. App maintenance costs were also assumed at 25% of the development cost per year. Intervention effects were estimated based on best available evidence from the literature deeming the intervention cost-effective in accordance with the UK reference case (19). In addition, a recent systematic review of dietary digital interventions concluded that mHealth interventions that are not cost-effective in the short-term may likely be cost-effective in the long-term due to cost-offsets and wider user reach (20).

In the absence of data, feasibility studies can provide insights into the suitability of study designs, methodological approaches, and economic outcomes (21). The HelpMeDoIt randomized controlled trial tested the feasibility and acceptability of evaluating a mobile dietary app designed for weight loss amongst adults with overweight and obesity through mobilizing social networks (22). Data collected for economic evaluation included NHS resource use, participant-borne costs (e.g., grocery shopping), interventions costs, health related quality of life (HRQoL) and capability wellbeing. App development and maintenance costs were valued, alongside quotes for future app maintenance (23). This is an important consideration given that app design and software features need to be regularly updated to maintain user engagement and app function (14). Although the study was not powered to detect significant changes, the intervention had potential to be effective, with modest decreases in BMI and sedentary time within the intervention group, thus generating moderate effect sizes.

Evaluations of health promotion apps are lacking (24). The Change4Life Food Scanner app was first released as part of a mass media campaign by Public Health England (PHE), a UK Government agency (25). The app aims to raise awareness on the nutritional content of packaged foods through a barcode scanner feature. The Food Scanner app contains a series of evidence based components designed to effectively change behavior, i.e., Behavior Change Techniques (BCTs) (26), with some evidence to suggest it is effective in improving dietary behaviors in the short term when evaluated as part of the wider Change4Life campaign (25). Little is known regarding whether the Change4Life Food Scanner app is cost-effective in improving dietary behaviors. This is important as the development of the app and its contents required substantial financial input and resources.

There is limited available data and guidance surrounding the economic evaluation of public health mobile apps. The ways in which economic models are produced can highly affect final cost-effectiveness results. In order to inform the evaluation of the Change4Life Food Scanner app and to subsequently design a mathematical economic model, an understanding of the decision problem needs to be formed that captures the varying perspectives of the system and the causal relationships between factors within the system that lead to short-term and long-term behavior change and associated outcomes.

The aims of this study were to (1) explore the feasibility of collecting cost and outcome data when evaluating the cost-effectiveness of the Food Scanner app; and (2) investigate whether randomized controlled trials offer a feasible approach to assessing whether the Food Scanner app is cost-effective in improving dietary choices. This was achieved through a multi-step process which firstly involved the engagement of stakeholders to design a conceptual model that would then inform the parameters of the feasibility study.

## 2. Materials and methods

### 2.1. Stakeholder engagement

Stakeholder engagement was carried out to inform the conceptual model of the Food Scanner app evaluation. This involved an interactive half-day workshop, and interviews for those unable to attend (one in-person interview with two stakeholders simultaneously and a single online video call) between November 2019 and January 2020. Participants were identified through available publications, existing networks and targeted decision makers working within policy. The total sample consisted of nine academics, two Government workers and one non-profit worker. Stakeholders had expertise within digital interventions, health economics and/or obesity research.

Stakeholders were provided with a draft version of a conceptual model that was informed by the existing behavior change literature, and which informed the methods of the feasibility study. The stakeholder event aimed to, (1) discuss factors that need to be assessed within dietary digital interventions; (2) explore current perspectives of the causal pathway by which a dietary app may lead to obesity prevention and improved health and wellbeing outcomes within a complex system; and (3) discuss potential issues and recommendations of evaluating the effectiveness and cost-effectiveness of dietary apps. Discussions involved mapping out the decision problem (i.e., revising the conceptual model), identifying the short-term and long-term priority outcomes for evaluation, and identifying resource use and associated costs of the Change4Life Food Scanner app from an intervention, user, healthcare and societal perspective. The conceptual model was then updated to reflect the stakeholders’ feedback.

**FIGURE 1:**
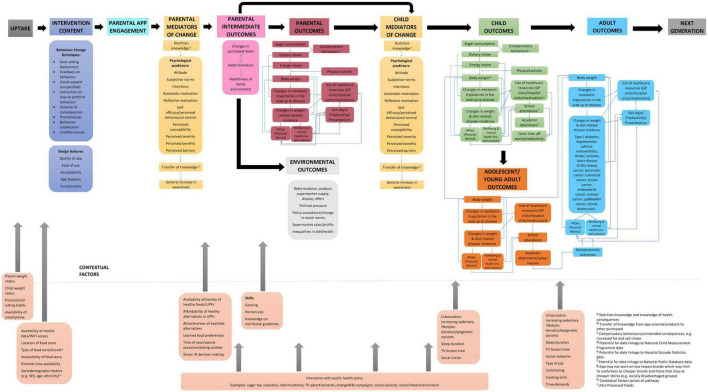
Conceptual model of the Change4Life Food Scanner app.

Stakeholders identified the pathways by which the Change4Life Food Scanner app impacts on dietary intake and childhood obesity prevention (see Figure 1). The model is split into two sections; the upper section describes the pathways to behavioral outcomes leading from app uptake, whilst the lower section describes contextual factors that may facilitate, or hinder behavior change success. The model begins with the provision of the Food Scanner app, which comprises of eight BCTs through which behavior is shaped (26). Alongside BCTs are app design features that are important to maintaining user engagement. Through using the app, users’ nutrition knowledge and psychological predictors of behavior change may improve, leading to a general increase in awareness of healthy diets. These are considered proximal outcomes.

Although intermediate outcomes are changes in behavior, they often precede the main desired effects. Within the model, changes in purchased items, habit formation, and healthiness of home environment are predicted to lead to parental outcomes, child mediators of change and environmental outcomes. Environmental outcomes are a result of the food system responding to consumer demands and changes in behavior. Parental and child outcomes describe how changes in sugar intake, lead to changes in dietary and energy intake, which may have an impact on body weight. These are considered medium-term outcomes, whilst environmental outcomes are considered distal.

Increases in body weight may lead to changes in metabolic trajectories in the lead up to disease, and changes in weight and diet-related disease incidence. In the long-term this is predicted to lead to increased use of healthcare resources, increased sick days off school or work, and a negative impact on physical and mental HRQoL and wellbeing. Childhood outcomes will continue into adolescence and will get worse into adulthood. These are considered distal outcomes.

Ideally, the Food Scanner app will lead to improvements in knowledge and awareness of nutrition in the short-term. This will lead to a decrease in sugar consumption and thus a reduction in total energy intake in the short to medium-term. This will then lead to a reduction in BMI in the medium-term, which will be protective of ill-health in the long-term. Contextual factors consider other aspects within the system that may facilitate or hinder behavior change. App engagement may interact with contextual factors and/or other policies within the system which may have additional positive impacts on behavioral outcomes.

### 2.2. Pilot and feasibility study

#### 2.2.1. Study design

Outcomes from the stakeholder engagement and conceptual model were used to inform trial design. The study was conducted as part of a pilot randomized controlled trial, which tested the feasibility, acceptability, and sustainability of evaluating the Change4Life Food Scanner app in reducing overall energy intake and sugar consumption in 4–11 years-old children through parental behavior change. Using a non-blinded parallel trial design, participants were randomized into an intervention condition or usual practice control condition in a 1:1 allocation ratio. A randomization sequence of 50 was produced at first using Microsoft Excel, with 20 sequences following thereafter per block (a total of four blocks). Random allocation sequence, participant enrollment and participant assignment to conditions was conducted by the study team.

The trial was registered in the Open Science Framework (27). Ethical approval was obtained by the University of Sheffield Research Ethics Committee (026380) in August 2019. The study adhered to the Consolidated Standards of Reporting Trials (CONSORT) for pilot and feasibility studies (28).

#### 2.2.2. Participants and recruitment

Recruitment took place between January and June 2020 in Yorkshire and the Humber region of the UK. The recruitment strategy included recruitment from primary schools. This occurred *via* school communication methods (e.g., signposting in school newsletters, SMS services, school app), and distribution of flyers provided by the study team to be sent home to parents. Online recruitment methods were also implemented, which included adverts distributed *via* the University’s mailing lists, online study recruitment, and social media platforms (Facebook and Twitter). A weblink directed interested volunteers to the online information sheet and consent form.

Participants were informed that the study was exploring parents’ views on dietary online programs or mobile apps. The eligibility criteria for participation included being a parent of a primary school child aged 4–11 years, owning a smartphone with data access and sufficient storage space, an active grocery shopper in the household or involved in child’s food provisions, grocery shopping dominantly undertaken at a grocery store or supermarket, not currently using the Change4Life Food Scanner app, and the child has no health condition that affects diet (excluding allergies), e.g., cystic fibrosis.

Upon study completion, participants received either a £35 (intervention) or £30 (control) shopping voucher for reimbursement of their time. In addition, participants who completed the study were entered into a prize draw for a £150 Virgin Experience Days gift card. As this was a feasibility study, participants who withdrew were contacted and asked to complete a short survey to detail reasons for withdrawing. To incentivize completing this survey participants were entered into a prize draw for a £20 Love2Shop gift voucher.

#### 2.2.3. Intervention and control

The intervention involved written contextual guidance on healthy eating behaviors obtained from Change4Life webpages, which prompted participants to download and use the Change4Life Food Scanner app to make healthier food choices and be a “sugar smart shopper.” Details of the app’s features and BCTs have been previously published (26). Briefly, the app encourages healthier food and drink choices by providing nutritional feedback of barcode scanned items through various visual methods. Sugar, salt, and saturated fat content is depicted in sugar cubes, salt sachets and fat slabs, alongside grams. Information, when available, is provided per 100 g/ml and per portion.

The control condition consisted of usual practice (no contextual information or guidance was provided regarding healthy eating behaviors and no reference was made to Change4Life).

### 2.3. Study procedures and measures

Upon consenting, participants completed sociodemographic measures which consisted of child age and sex, child and parent height and weight, location, parent ethnicity, parent education and household size and income. Data on household income was used to group participants on level of economic deprivation based on the Index of Multiple Deprivation (29). Participants were then randomized into an intervention or control arm. All participants completed 3 days food diaries *via* myfood24^®^ (30), and psychosocial and health economic measures *via* online surveys (Qualtrics, Provo, UT, United States) (31) at baseline and 3 months follow up. Only after completion of baseline measures did participants in the intervention arm receive intervention exposure. Those in the intervention arm completed app engagement measures fortnightly over 12 weeks.

At 3 months follow-up, participants completed 3 days food diaries using myfood24^®^, psychosocial measures and health economic measures as previously described. In addition, participants provided study and app feedback through open- and closed-ended questions.

The duration of the study was bounded by time constraints of the project. Details of the study, including feasibility, acceptability, and clinical efficacy outcomes, will be published elsewhere (manuscript in preparation). This paper reports the feasibility of collecting economic outcomes of the intervention for the purposes of cost-effectiveness analysis.

### 2.4. Economic study and statistical methods

A cost-consequence analysis was conducted. Cost-consequence methods have been recommended for the evaluation of digital products (32). These consisted of healthcare resource use and associated costs, school absence, workplace absenteeism, and HRQoL measures. Statistical analysis was carried out on STATA/SE 15.1. This study undertook a healthcare and societal perspective to address the generalizable issues of feasibility pertaining to both. Questions were adapted from a number of surveys identified from the Database of Instruments for Resource Use Measurement (DIRUM), (33) except for HRQoL measures. Permissions were obtained from the copyright holders of original surveys.

Despite economic impacts of the Food Scanner app being reflected as distal outcomes within the conceptual model, these were investigated within this study to assess the feasibility of using such measures within a future cost-utility and/or cost-effectiveness analysis of the Food Scanner app. In addition, as this is a feasibility study, and therefore not powered to detect significant differences, descriptive statistics were conducted only, and inferential statistics were not. Reported comparisons need to be interpreted with caution in all cases, and mean differences are reported trends only.

#### 2.4.1. Study and intervention costs

The majority of study costs were related to the completion of food diaries using myfood24^®^. Costs relating to the production of physical resources were not factored into cost estimates as they were considered sunken costs (a cost spent that cannot be reversed). With regards to opportunity costs associated with the distribution of physical resources, this was also not considered given that distribution of trial promotion material was no longer actioned by schools and community centers due to COVID-19 lockdown measures. This also meant that the trial incurred cost losses incurred by printing and postage services of materials that were not distributed to parents due to lockdown measures.

Separate to trial data, a Freedom of Information request was submitted by the study team to PHE in October 2020 enquiring about the total costs of the Change4Life campaign, as well as development and maintenance costs of the Change4Life Food Scanner app. This was submitted to estimate intervention costs as data was not available publicly. Access to such data would allow us to conduct more accurate cost-effectiveness analyses going forward and would allow the estimation of the mean cost per user (15). A response was received in December 2020 outlining total marketing costs associated with the Change4Life campaign. In addition, to gain insight into the cost per download, the Change4Life Food Scanner app webpages were consulted for number of downloads for both Google Play and the Apple App store (34, 35).

#### 2.4.2. Health related quality of life

Participants completed the Child Health Utility 9 Dimension (CHU9D) instrument, a short validated pediatric HRQoL instrument (36, 37) which was used a measurement of health outcomes within this study. This is a preference-based measure designed for self-completion by 7–17 years-olds and proxy completion for younger age groups (38). Given that parents were the ones participating in the trial, the parent proxy version was utilized. The instrument consists of nine dimensions: worried, sad, pain, tired, annoyed, schoolwork/homework, sleep, daily routine, and ability to join in activities. Each dimension consists of five response options ranging from the least severe option (e.g., my child does not feel worried/sad/tired today) to most severe (e.g., my child feels very worried/sad/tired today). Parents are asked to decide which option represents their child best on the day of completion. Utility values (value or preference that the population gives to a particular health state) were calculated through the use of UK adult preference weights (i.e., utility values were based on UK adult preferences) (39, 40). Utility values were then used to calculate quality adjusted life years (QALYs) using the trapezium rule (area under the curve; a measure of effect) (41). The CHU9D was used to assess the feasibility of collecting HRQoL measures when evaluating a dietary mobile app.

#### 2.4.3. Child healthcare use

Current evidence indicates increased healthcare use and hospital admissions (42) and costs amongst children with overweight and obesity (43). As such, this study tested the feasibility of collecting self-reported healthcare resource usage as a basis for measuring healthcare costs. Participants were asked to report healthcare services used in the last 3 months including number of visits and total length of time per contact (44); These questions were included in order to assess incremental effects of the Food Scanner app on short term health resource use. Healthcare resource costs, including general practitioner (GP), nurse, dental, hospital inpatient and hospital outpatient were estimated using 2021 PSSRU unit costs (45). The National Schedule of NHS Costs (year 2019/2020) was used to estimate accident and emergency costs (46). See Table 1 for healthcare cost data and assumptions.

**TABLE 1:** Healthcare resource costs and assumptions.

Resource	Cost (£)	Unit	Assumption
GP consultation	3.70	Min	GP costs were estimated at £3.70 per minute of patient contact, including qualification costs. This excluded direct care staff costs as the majority of the trial ran during the COVID pandemic, and the majority of GP consultations had become *via* telephone.
Nurse	0.73	Min	Dental costs were estimated at 73.3p per minute of patient contact (based on £44 per hour). Costs included qualifications.
Hospital inpatient	827	Visit	Inpatient costs are not calculated by time. Costs were available for non-elective short and long stays. Given that only one respondent had an inpatient stay which lasted less than 24 h, it was considered a short stay.
Hospital outpatient	137	Visit	Outpatient attendance was not available by minutes or hours, but rather having occurred or not, despite this information being collected from participants. Given that no further details were collected regarding the nature of the outpatient visit, a weighted average cost of all outpatient attendances was selected.
Accident and emergency	182	Visit	Accident and emergency costs were sourced through the National Schedule of NHS Costs 2019–2020 for NHS trusts and NHS foundation trusts. Data was not collected on the reason for the A&E visit, and whether participants were admitted, if they had any investigations or treatments. Therefore, a weighed mean average of all A&E visits was selected, accounting to £182 per unit.
Non-routine dental	3.28	Min	Dental costs were estimated at £3.28 per minute of patient contact (based on £197 per hour of patient contact). Data on the nature of the appointment was not collected therefore whether any dental procedures were carried out can not be ascertained.

All costs were sourced through the Personal Social Services Research Unit (PSSRU) 2021 Database, unless otherwise stated.

#### 2.4.4. Productivity and personal financial losses

Societal perspectives include costs which matter to society, such as workplace productivity losses and personal financial losses. Outcome measures considered school absenteeism in the past 3 months due to a health problem (47) and workplace absenteeism in the past 3 months due to child’s health (48). Productivity losses were estimated by multiplying days off work due to child health by median daily rate of £108.20, based on the Sheffield median weekly income (49). Increases in grocery shopping expenditure can be an unintended consequence of dietary interventions (50, 51) given that healthier foods are more costly than less healthier alternatives (52, 53). In order to determine whether a full investigation into grocery expenditure is warranted in a full-scale trial, participants in the intervention arm were asked at 3 months follow up, “using the Food Scanner app has led me to spend… a lot less/slightly less/the same/slightly more/a lot more… on groceries.”

#### 2.4.5. Sensitivity analysis and handling of missing data

It is not unusual for cost data to be right skewed or follow a gamma distribution, as opposed to a normal distribution. This is due to the majority of the population being in good health, therefore incurring minimal healthcare costs (54). Standard deviation z-scores were explored for healthcare and workplace absenteeism cost data (i.e., productivity costs). Extreme data points, interpreted as those five standard deviations from the mean, were removed from the analysis, as part of a sensitivity analysis.

In addition to complete case analysis, multiple imputation (MI) was also conducted as part of a sensitivity measure. It allowed us to explore the feasibility of using such approaches when evaluating the economic impacts of a dietary app, especially when retention rates could impact on the completeness of data.

Multiple imputation methods were adopted using Monte Carlo simulation techniques (55). The Gaussian normal regression imputation method was conducted, where data was assumed missing at random (MAR). Sociodemographic data with complete cases were selected as auxiliary variables for MI purposes. These included: condition, child age, child sex, ethnicity, location, education, household income and household size. Therefore, participants with missing sociodemographic data were removed from the dataset for multiple imputation purposes (*n* = 12). These respondents did not report any school absences, workplace absenteeism or healthcare resource use that could lead to noticeable changes in total costs and mean differences.

Variables considered for MI included QALYs (calculated from CHU9D outcomes), healthcare resource costs, workplace absenteeism due to child’s health, and school absenteeism, all at baseline and 3 months follow up. All these variables had between 35 and 50% missing data. The percentage of missing cases per variable determined the number of imputations per variable (56). Additional imputations were conducted in cases where the Fraction of Missing Information (FMI) percentage was above the number of imputations. A single result per case was calculated based on the average value of imputations per variable. Multiple imputation was favored over other missing data handling techniques as it considers the variance between and within variables and reduces chances of biased estimates which often arise in other methods (57).

## 3. Results

### 3.1. Participants

**FIGURE 2:**
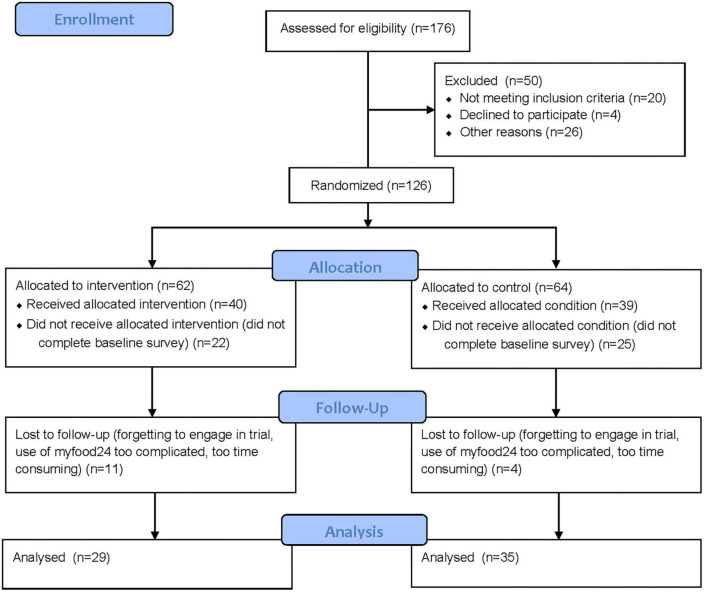
Consort flow chart of the Change4Life Food Scanner app pilot and feasibility trial.

A total of 176 participants were assessed for eligibility through a screening questionnaire. Of which, 50 were excluded from further participation in the study. Reasons included not meeting the inclusion criteria, not providing an email address to forward trial material, and not fully completing the consent form. As such, 126 (72%) participants were randomized to the intervention (*n* = 62) or control arm (*n* = 64). In the intervention arm, 40 (65%) completed baseline measures and therefore received the allocated intervention; whilst 22 (35%) participants did not engage in the study material. In the control arm, 39 (61%) participants completed baseline measures and 25 (39%) did not engage in the study material. At 3 months follow up, data was analyzed from 29 (47%) participants in the intervention and 35 (55%) in the control arm (see Figure 2).

Table 2 outlines the baseline characteristics of the study sample. Overall, the sample consisted of parents of children with an average age of 6.81 (SD = 2.04) and a similar distribution of male and females. The parent sample was predominantly White British (71%). The majority of parents had completed higher education (69%). Data on household income suggested that 32% of the sample were in the least deprived quintile, whilst 13% were in the most deprived. Most of the sample had a household size of four or smaller (83%).

**TABLE 2:** Baseline characteristics of randomized participants.

		All	Intervention	Control
*N*	–	126	62	64
Missing cases	–	12^a^	7^b^	5^c^
Child age (years)	Mean (SD)	6.81 (2.04)	6.77 (1.77)	6.85 (2.28)
Child sex	*N* (%) Female	60 (51.7)	26 (46.4)	34 (56.7)
	*N* (%) Male	56 (48.3)	30 (53.6)	26 (43.3)
Parent ethnicity	*N* (%) White British	81 (71.1)	41 (75.4)	40 (67.8)
	*N* (%) White other	9 (7.9)	5 (9.1)	4 (6.8)
	*N* (%) Asian	11 (9.6)	4 (7.3)	7 (11.9)
	*N* (%) Mixed White and Black	4 (3.5)	3 (5.5)	1 (1.7)
	*N* (%) Other	9 (7.9)	2 (3.6)	7 (11.9)
Parent education	*N* (%) Higher education^d^	79 (69.3)	39 (70.9)	40 (67.8)
	*N* (%) Other	35 (30.7)	16 (29.1)	19 (32.2)
Household income (quintiles)	*N* (%) Q1—most deprived	16 (12.7)	10 (16.1)	6 (9.4)
	*N* (%) Q2	5 (4)	2 (3.2)	3 (4.7)
	*N* (%) Q3	16 (12.7)	6 (9.7)	10 (15.6)
	*N* (%) Q4	28 (22.2)	14 (22.6)	14 (21.9)
	*N* (%) Q5—least deprived	40 (31.7)	18 (29.0)	22 (34.4)
	*N* (%) Unknown^e^	21 (16.7)	12 (19.4)	9 (14.1)
Household size	*N* (%) 2	10 (8.8)	6 (10.9)	4 (6.8)
	*N* (%) 3	32 (28.1)	9 (10.9)	23 (39.0)
	*N* (%) 4	53 (46.5)	33 (60)	20 (33.9)
	*N* (%) 5	14 (12.3)	4 (7.3)	10 (16.9)
	*N* (%) Other	5 (4.4)	3 (5.4)	2 (3.4)

^a^10 missing cases for variables: age, sex.

^b^Six missing cases for variables: age, sex.

^c^Four missing cases for variables: age, sex.

^d^Defined as higher education qualification below degree level, degree level qualification, or a Masters/Ph.D. or equivalent.

^e^Includes missing and unknown cases.

### 3.2. Study costs

The total cost of the feasibility study was £4666.29 in year 2020 (Table 3). The average cost was calculated at £36.05 (2020) per participant (*n* = 126). The cost almost doubles to £70.98 (2020) per participant when numbers are based on study completers (*n* = 64).

**TABLE 3:** Feasibility trial costs.

Item	Cost
Myfood24^®^ —2 years access+participant entries	£1810
Incentives—gift vouchers (intervention)	£1015
Incentives—gift vouchers (control)	£1050
Incentives—withdrawal survey voucher	£25
Incentives—prize draw (Virgin Experience Days Gift card)+shipping	£154.99
Mobile sim card	£44.90
Social media advertising	£419
Call for Participants advertising	£24
Print and postage services	£123.40
Total	£4666.29

### 3.3. Intervention related costs

Data from Google play shows that the Change4Life Food Scanner app has achieved over 500,000 downloads to date (34). This information is not available on the Apple app store. Outcomes from the FOI request noted that PHE agrees to a fixed rate for services, but no further information or breakdown of costs was provided regarding development and maintenance costs. The FOI request was therefore unsuccessful in gaining the information necessary for a comprehensive cost-consequence analysis. On the other hand, PHE confirmed they had run two Change4Life campaigns in 2017 encouraging healthy eating for children and families, to the value of £3.5 million in paid media activity. As part of these campaigns, consumers were encouraged to download the “Be Food Smart” app (as the Food Scanner app was then called) to find out how much sugar, fat and salt were in a range of popular products, and to help consumers choose healthier options. PHE further confirmed that they do not hold any information on the Return on Investment for the Change4Life campaign, or the Food Scanner app. As we were unable to retrieve specific app-related costs, cost per download could not be quantified.

When investigating the financial consequences of using the app, 20 out of 28 participants (71%) reported that using the Food Scanner app led them to spend the same amount on groceries. Whereas seven participants (25%) reported that using the app led them to spend slightly more on groceries. Only one participant reported spending less on groceries after using the app (3.6%).

### 3.4. Health related quality of life

A total of 78 (62%) participants completed CHU9D measures at baseline, and 63 (50%) completed these measures at follow up. One participant was removed from analysis at 3 months follow up due to missing data. This resulted in 62 complete cases across baseline and follow up. Very few problems were reported in children’s HRQoL (see Supplementary Table 1). Similar mean scores were found between baseline and follow-up across all dimensions for both intervention and control groups. Finally, there was a greater range, in the direction of worse HRQoL at follow-up, in comparison to baseline, for the intervention group only.

Table 4 outlines mean differences (SD) between baseline and follow-up across conditions. The mean difference (SD) for the total CHU9D score at follow-up was –0.464 (4.558) for the intervention arm and –0.588 (4.054) for the control arm. When CHU9D scores were converted into utilities, the mean difference at follow-up was 0.007 (0.104) for the intervention arm, and 0.014 (0.089) for the control arm. This resulted in 0.222 QALYs for children in the intervention arm (SD = 0.019, 95% CI: 0.215; 0.230) and 0.226 QALYs (SD = 0.016, 95% CI: 0.220; 0.232) in the control arm over the 3 months period of the study. This amounted to a mean reduction in QALYs between groups over the trial period of –0.004 (SD = 0.024, 95% CI: –0.005; 0.012).

**TABLE 4:** Costs (£) and consequences related to intervention and control conditions.

Costs and consequences	Intervention	Control
**Child healthcare costs (£)**		
*N*	26	32
Mean difference (SD) between baseline and follow-up	–52.560 (213.59)	–21.790 (87.91)
95% CI	–138.83; 33.71	–53.48; 9.90
**Health related quality of life score^a^**		
*N*	28	34
Mean difference (SD) between baseline and follow up	–0.464 (4.558)	–0.588 (4.054)
95% CI	–2.232; 1.303	–2.003; 0.826
**Utility score**		
*N*	28	34
Mean difference (SD) between baseline and follow up	0.007 (0.104)	0.014 (0.089)
95% CI	–0.0336; 0.0471	–0.0169; 0.045
**Quality adjusted life years**		
*N*	28	34
Mean (SD) between baseline and follow up	0.222 (0.019)	0.226 (0.016)
95% CI	0.215; 0.230	0.220; 0.232
**School absenteeism**		
*N*	29	32
Mean difference (SD) between baseline and follow-up	–0.362 (1.253)	–0.547 (1.364)
95% CI	–0.839; 0.114	–1.039; –0.055
**Workplace productivity due to child’s health (£)**		
*N*	27	34
Mean difference (SD) between baseline and follow-up	–80.148 (235.516)	–15.912 (54.148)
95% CI	–173.315; 13.019	–34.805; 2.981

^a^Based on the Child Health Utility 9 Dimension instrument.

### 3.5. Child healthcare use

Parents reported more frequent healthcare resource use at baseline compared to follow-up within both study arms (see Table 5). GP services were most frequently reported. There was greater healthcare resource use and associated costs at baseline compared to follow-up in both study arms. There was a £1684.30 decrease in healthcare costs at follow-up in the intervention arm, and £782.31 decrease in the control arm over the 3 months study period. As outlined in Table 4, mean difference (SD) between baseline and follow-up child health-care costs was –£52.56 (95% CI: –£138.83; £33.71) for the intervention arm (*n* = 26) and –£21.79 (95% CI: –£53.48; £9.90) for the control arm (*n* = 32). This amounted to a mean reduction between groups over the data collection period of –£30.77 (SD = 230.97; 95% CI: –£113.80; £52.26).

**TABLE 5:** Total healthcare resource use and associated costs.

Healthcare resource	Intervention		Control	
	**Baseline (*n* = 38)**	**Follow up (*n* = 28)**	**Baseline (*n* = 37)**	**Follow up (*n* = 33)**
**Healthcare resource use (minutes)^†^**				
GP	85	20	75	32
Nurse	0	0	15	5
Hospital inpatient	840	0	0	0
Hospital outpatient	55	25	45	40
A&E	60	0	625	0
Non-routine dental	80	90	51	15
Total	1,120	135	811	92
**Healthcare resource use (visits)^†^**				
Hospital inpatient	1	0	0	0
Hospital outpatient	2	2	2	2
A&E	1	0	2	0
**Healthcare resource costs (£)^§^**				
GP	388.5	74	277.5	192.4
Nurse	0	0	18.33	0
Hospital inpatient	827	0	0	0
Hospital outpatient	274	274	274	274
A&E	182	0	364	0
Non-routine dental	656	295.2	364.08	49.2
Total	2327.50	643.20	1297.91	515.60

^†^Calculated as the sum of the number of visits x average appointment time per participant.

^§^Calculated as healthcare resource use x cost of healthcare service (see Table 1). In cases where healthcare visits are valued per unit costs, this was quantified by number of visits x healthcare service cost.

### 3.6. Productivity and personal financial losses

Total days off school due to ill health, and consequential parent time off work, over the past 3 months was reported (see Table 6). Over the trial period, there was a reduction of 20 days off work in the intervention arm, and a reduction of 6 days off work in the control arm. Baseline absenteeism cost amounted to £2272.20 within the intervention arm, and £649.20 within the control arm. At 3 months follow up, workplace absenteeism costs amounted to £108.20 in the intervention arm and £0 in the control arm.

**TABLE 6:** Productivity losses.

Absenteeism and associated costs	Intervention		Control	
	**Baseline (*n* = 40)**	**Follow up (*n* = 27)**	**Baseline (*n* = 38)**	**Follow up (*n* = 35)**
Child total days off school due to ill health	14.5	4	19.5	0
Parent total time off work due to child health	21	1	6	0
Parent productivity costs (£)^†^	2272.20	108.20	649.20	0

^†^Cost of paid time off work due to child’s health (total days off by median daily rate £108.20 based on Sheffield median weekly rates).

Based on complete case analysis, mean difference between baseline and follow-up school absenteeism was –0.362 (95% CI: –0.839; 0.114) per child for the intervention arm (*n* = 29) and –0.547 (95% CI: –1.039; –0.055) for the control arm (*n* = 32). This amounted to a mean difference reduction of –£80.15 (95% CI: –£173.315; £13.019) in workplace productivity losses within the intervention arm and –£15.91 (95% CI: –£34.81; £2.98) in the control arm per participant. This resulted in a mean difference reduction of –£64.24 (SD = 241.66, 95% CI: –£147.54; £19.07) between study arms at follow up.

### 3.7. Sensitivity analysis

Two data points were removed from the analysis due to *z*-scores greater than five. Mean differences (SD) between baseline and follow-up child healthcare costs were –£14.28 (95% CI: –£50.89; £22.33) for the intervention arm (*n* = 25) and –£21.84 (95% CI: –£53.55; £9.87) for the control arm (*n* = 32). This amounted to a mean difference between groups over the data collection period of £7.56 (SD = 124.91; 95% CI: –£39.66; £54.70). There was a mean reduction (SD) between baseline and follow-up workplace productivity costs of –£41.62 (95% CI: –£92.70; £9.47) for the intervention arm (*n* = 26) and –£15.88 (95% CI: –£34.74; £2.98) for the control arm (*n* = 34). This amounted to a mean difference between groups over the data collection period of –£25.73 (SD = 137.54; 95% CI: –£73.98; £22.51).

The number of missing observations that were accounted for within multiple imputation ranged between 39 and 42 at baseline, and 54–55 at 3 months follow up. The dataset comprised of 114 complete observations after multiple imputation (intervention: *n* = 55; control: *n* = 59). Supplementary Table 2 provides a breakdown of totals and means of multiple imputation outcomes. Mean differences between baseline and follow-up of multiple imputation cost and consequence outcomes are outlined in Supplementary Table 3. In summary, mean differences between study conditions over the study period led to a mean decrease in healthcare resource costs by –£12.95 (SD = 163.92, 95% CI: –£55.49; £29.59), workplace productivity cost reduction of –£36.72 (SD = 174.12, 95% CI: –£81.74; £8.31), and a mean reduction in QALYs by –0.005 (SD = 0.018, 95% CI: 0.000; 0.009, see Supplementary Table 3).

## 4. Discussion

The current pilot study investigated the feasibility of collecting and evaluating cost-effectiveness measures to help inform the development of a full-scale trial evaluating the Change4Life Food Scanner app. This is the first study, to our knowledge, to assess the cost and associated consequences of a UK Government dietary app. All analyses are preliminary and should be interpreted with caution. Complete case analysis suggested a reduction in healthcare resource costs, school absence and workplace productivity losses, and a modest increase in utilities, at follow-up, for both intervention and control arms. When mean differences were compared between groups, there was a greater reduction in both healthcare expenditures and productivity losses in the intervention arm, alongside a modest reduction in QALYs. Similar findings were apparent within multiple imputation. These findings suggest that the Food Scanner app may have the potential to be cost-saving from a healthcare and societal perspective, however, a larger sample size is needed to test for significance between-groups, alongside a longer follow-up period to ascertain intervention effects on distal outcomes.

The time horizon of the study was considerably short for the outcomes under investigation. Overweight and obesity alongside healthcare and societal consequences are long-term trajectory issues that cannot be validly predicted from this 3 months feasibility study. The presence of a long-term economic model would provide the basis for making predictions about the long-term impact of short-term changes observed in this study and a full-scale trial. Therefore, we cannot ascertain whether the Food Scanner app will have any impacts on HRQoL, healthcare and societal costs in the long-term, as suggested within the conceptual model. A full-scale trial with a 24 months follow-up period may be necessary to allow for any short- (e.g., diet) and medium-term (e.g., body weight and HRQoL) impacts of the intervention to be captured.

Economic evaluations alongside trials involve an analysis of trial costs. Costs of running the feasibility study amounted to £36.05 per participant, based on the number of consenting participants. However, costs per participant almost doubled when the average is based on study completers. Alongside sample size calculations, such costing will provide an estimate on the funding requirements of a full-scale trial. Calculation of study costs could be used to inform a full pre-trial model analysis to calculate the expected net benefit of a full trial design and whether this is positive or negative. However, to achieve this, intervention costs estimates would be needed alongside a long-term impact model. The latest Medical Research Council (MRC) guidance on the evaluation of complex interventions has suggested that economic modeling could be adopted within feasibility studies to verify whether the predicted benefits of the intervention justify both intervention costs and that of any future research (i.e., expected value of perfect information analysis) (58). This could help determine whether the implementation of a full-scale trial is beneficial.

The current study was unable to account for costs relating to the development and maintenance of the Change4Life Food Scanner app and attempts to access this information were unsuccessful. This was partly due to the costs of the app being intertwined with the costs of running the broader Change4Life campaign. In addition, there is a lack of information in the public domain regarding total number of previous and current app installs. There is a misconception that apps are a low-cost approach to achieving public health outcomes (12). Whilst the cost per download is low, and some apps are available for free to the user, the costs of development and ongoing maintenance, as well as the program or campaign in which they are embedded, are substantial (14). For example, Kalita et al. (18) evaluated a multicomponent intervention that included a dietary app component. App development costs (expert estimation) was estimated at £324,000, for an app duration of 10 years, in addition to 5 years of development time. Maintenance costs were assumed to be 25% of app development costs, amounting to £16,200. On the other hand, Tully et al. (24) estimated app development costs at approximately €11,000, whilst maintenance costs were estimated at approximately €2000 (15–20% of app development costs). Additional costs were also flagged, such as cloud data storage).

Alongside substantial app costs, there is difficulty in demonstrating intervention effects. This includes short-term intervention effects, which are both small and difficult to measure, as well as long-term effects, due to difficulty in providing validated approaches to predicting long term outcomes (59). Therefore, economic evaluation is imperative to gain estimates of long-term outcomes that otherwise would not be possible. Given the difficulties in external evaluation, and more importantly in light of accepted frameworks for evaluation of complex interventions in complex settings (58), economic evaluations and long-term modeling should be embedded within programs. However, further transparency and research is needed exploring app development and maintenance costs by intervention complexity and features in order to guide evaluations. Such research may consider the inclusion of app developers as key stakeholders within discussions whereby a map of the app development journey can be mapped out alongside cost estimates. However, it is also likely that the size of app development companies and location may impact on cost of services. Such data will help guide the estimation of app-related costs in the absence of data and should be utilized alongside a series of sensitivity analyses.

App promotion is a necessary driver to maximize app uptake and therefore has the potential to increase cost-effectiveness of app-based interventions (14). Given that the Food Scanner app was initially released as part of a multi-media national campaign comprising of billboard and TV-based advertisements, as well as resources for schools (25), calculations of app-related costs may become entangled with Change4Life promotion material and general campaign costs. Cost-effectiveness of app promotion has been previously investigated within evaluations. A conceptual model was produced to reflect the likely population of New Zealand that would download a promoted weight loss app and use it at least once. Results suggested that smartphone app promotion costs amounted to NZ $2,883,000 over 1 year, resulting in small health gains and borderline cost-effectiveness at a population level. However, the model did not factor in app use by those not exposed to the mass media campaign, as well as duration and quality of app engagement (60, 61). In the case of the Food Scanner app, costs associated with the Change4Life campaign in general were available only. Using these cost-estimates within cost-effectiveness analysis of the Food Scanner app risks overestimating costs involved in relation to the intervention received. Given that the Food Scanner app is freely available on the app market, individuals may engage with the app without having been exposed to, or engaged with, any of the other campaign material. Although the Food Scanner app can be considered as a standalone intervention, it is ultimately a component within a larger complex intervention (or campaign) operating in a complex obesity system. Ideally, complex interventions alongside their components should be evaluated individually to gain insight into the active ingredients leading to changes in behavior (58, 62).

Healthcare resource use, and associated costs, was reported throughout the trial period. Results suggested a greater reduction in healthcare expenditure within the intervention arm. We cannot ascertain whether such changes were due to intervention exposure given the short-term follow-up of the intervention, as any impacts on healthcare use are more likely to be distal as suggested within the conceptual model. In addition, the running of the trial was impacted by the coronavirus pandemic. The pandemic resulted in decreased population A&E attendance (63), and decreased outpatient services (64), therefore caution must be taken when interpreting results. Number of missing data for healthcare resource use measures were similar to other outcomes obtained within the trial. Although these measures were considered feasible, assumptions were made when costing the use of healthcare resources, given the ample costing options available on the National Schedule of NHS Costs 2019–2020 for NHS trusts and NHS foundation trusts, especially for A&E and inpatient services (46).

The CHU9D instrument was considered a feasible HRQoL measure for the purposes of the trial. Given the current study was only 3 months, we did not expect to see any considerable change in CHU9D outcomes, as was evidenced within our findings. Results suggested some worsening of HRQoL outcomes, though minimal, within the intervention group at follow-up. Given that COVID-19 was a study confounder, the pandemic may have impacted negatively on child outcomes and mental health (65). On the other hand, the lack of variability in CHU9D responses could suggest that the CHU9D is not sensitive enough to detect changes in HRQoL in a predominantly healthy sample. For example, a systematic review investigating utility values for childhood obesity interventions found very small but significant differences by child weight status (66). A longer study follow-up period, with a larger sample size, would help provide clarity regarding the CHU9D’s suitability, particularly if the intervention were to result in improvements in dietary choices.

There was a reduction in productivity losses at follow up, in both condition arms. These results are aligned with school absence data. Our measures did not account for whether time off work was taken as paid (annual leave) or unpaid leave. This ought to be considered in future revisions of trial measures, as it may risk overestimating productivity losses. Future revisions of this measure should also consider workplace absenteeism for both parents as opposed to the participating parent only, to account for differences in how responsibilities are divided within households. A recent review on the use of productivity loss instruments has recommended the use of the institute for Medical Technology Assessment MTA Productivity Cost Questionnaire to capture absenteeism, presenteeism and unpaid work over a 4 weeks recall period (67); which has been previously advised for increased recall precision (68). In addition, given that recruitment specifically took place in Yorkshire and the Humber, differences in median weekly wages by geographic region was not incorporated within costing assumptions. However, this may be necessary within a full-scale trial should recruitment be expanded to the UK more generally.

Dietary interventions may risk unintended economic consequences, which may act as a barrier to continued engagement or dietary behavior change (51). Approximately a quarter of the sample in the intervention arm reported having spent slightly more on groceries due to their use of the Food Scanner app. This is similar to previous research that aimed to improve the healthiness of children’s lunchboxes, however, resulted in a non-significant increase in the cost of packed lunches at follow-up (16). Given that a small proportion of individuals within the intervention arm reported increased grocery expenditures due to the 3 months trial, future measures within a full-scale trial ought to quantify these findings, for example through the collection of shopping receipts. This method has previously been used to monitor food purchasing behaviors (69).

Sensitivity analyses were conducted within the trial. Removal of outliers, or extreme data points, for cost data resulted in smaller mean differences between intervention and control arms over the trial period, in comparison to complete case analysis. Results suggested greater productivity losses within the intervention arm, as was the case within complete case analysis. However, after sensitivity analysis greater healthcare resource costs were found within the control arm, which was not the case within complete case analysis. Excluding outliers has demonstrated an impact on cost data. A future trial protocol should consider how outliers are to be interpreted and how extreme cost items should be handled. Previous research has adopted bootstrapping techniques, which reduces the impact of highly skewed data and extreme data points (70). Alternatively, the 95th percentile of the overall sample’s baseline and follow-up costs have also been used to determine cost outliers (71).

The current evaluation has considered a broad range of economic measures which were considered feasible and explored multiple imputation methods for missing data handling. However, the study did have several limitations. Opportunity costs for lost time for using the Food Scanner app was not accounted for. Given that data on time spent engaging with the app was collected, opportunity costs could have potentially been quantified. However, there would have been uncertainty regarding appropriate costing units. Another limitation involved the considerable amount of missing data, amounting to approximately 50% due to the high dropout rate early in the trial (before randomization exposure). Despite this, the sample size was still within the suggested range for pilot and feasibility studies (72, 73). However, there were considerable differences in baseline reported outcomes for healthcare resource use and parent time off work due to child health between study arms. We cannot ascertain whether differences in baseline characteristics may be driving differences in outcomes at follow up, as opposed to the intervention. It is necessary that participant retention methods are considered for a full-scale trial, alongside efforts to over-recruit participants to account for a high drop out.

This pilot and feasibility study exploring the economic and health impacts of the Change4Life Food Scanner app adds to the modest yet growing literature on the cost-effectiveness of mHealth dietary interventions. This is currently an under researched area, given the development and evaluation of dietary interventions has only started to emerge over the past decade. As such, the consideration of appropriate economic outcome measures, in addition to clinical outcomes, is necessary within feasibility studies before they are implemented in large-scale trials. Our results suggested that outcomes under investigation were feasible, though may require some minor revisions to best capture accurate data. The use of an RCT study design was also considered feasible to investigate the study question. However, given the nature of complex interventions within complex food systems (74), such designs may need to be supplemented with qualitative data collection to help explain the relationships between intervention exposure and outcomes of interest (75). In addition, in cases where missing data cannot be prevented, multiple imputation methods were considered a successful approach to handle missing data whilst considering both within- and between-participant variability. However, further research is warranted into the effectiveness of dietary smartphone apps, dietary app uptake, duration of use and the variability of costs associated with the development and ongoing maintenance of dietary apps.

## Data availability statement

The raw data supporting the conclusions of this article will be made available by the authors upon reasonable request, without undue reservation.

## Ethics statement

The studies involving human participants were reviewed and approved by the University of Sheffield Research Ethics Committee (026380) in August 2019. The patients/participants provided their written informed consent to participate in this study.

## Author contributions

SM conceived and managed the project, undertook data collection, data analysis, and data interpretation. All authors participated in the design of the study, critically reviewed the manuscript, reviewed, and accepted the final version of the manuscript.
