# Supplementary material for: Economic and health impacts of the Change4Life Food Scanner app: Findings from a randomized pilot and feasibility study
Source: Front Nutr. 2023 Mar 16;10:1125542. doi: 10.3389/fnut.2023.1125542 (PMC10061026; doi:10.3389/fnut.2023.1125542)
Supplement: Supplementary file 1 [file Data_Sheet_1.docx]

Supplementary Material

| **Supplemental Table 1**. **Parent-reported child health-related quality of life outcomes** | | | | |
| --- | --- | --- | --- | --- |
| **CHU9D** | **Intervention (n=28)** | | **Control (n=34)** | |
|  | **Baseline** | **Follow up** | **Baseline** | **Follow up** |
| **Total CHU9D** |  |  |  |  |
| Mean (SD) | 13.607 (3.521) | 13.143 (4.361) | 13 (3.384) | 12.412 (3.377) |
| Median | 13 | 11.5 | 12.5 | 12 |
| Range | 9-22 | 9-26 | 9-21 | 9-22 |
| **Worried** |  |  |  |  |
| Mean (SD) | 1.71 (1.117) | 1.32 (0.670) | 1.65 (1.041) | 1.44 (0.746) |
| Median | 1 | 1 | 1 | 1 |
| Range | 1-5 | 1-3 | 1-5 | 1-4 |
| **Sad** |  |  |  |  |
| Mean (SD) | 1.5 (0.793) | 1.21 (0.568) | 1.29 (0.719) | 1.35 (0.734) |
| Median | 1 | 1 | 1 | 1 |
| Range | 1-4 | 1-3 | 1-4 | 1-4 |
| **Pain** |  |  |  |  |
| Mean (SD) | 1.14 (0.356) | 1.07 (0.262) | 1.09 (0.288) | 1.09 (0.379) |
| Median | 1 | 1 | 1 | 1 |
| Range | 1-2 | 1-2 | 1-2 | 1-3 |
| **Tired** |  |  |  |  |
| Mean (SD) | 1.86 (0.932) | 1.89 (0.832) | 1.68 (0.878) | 1.56 (0.746) |
| Median | 2 | 2 | 1 | 1 |
| Range | 1-4 | 1-4 | 1-4 | 1-4 |
| **Annoyed** |  |  |  |  |
| Mean (SD) | 1.75 (0.928) | 1.71 (0.976) | 1.65 (0.849) | 1.59 (0.925) |
| Median | 1.5 | 1 | 1 | 1 |
| Range | 1-4 | 1-4 | 1-4 | 1-4 |
| **School work** |  |  |  |  |
| Mean (SD) | 1.61 (1.1) | 1.57 (1.069) | 1.32 (0.475) | 1.41 (0.783) |
| Median | 1 | 1 | 1 | 1 |
| Range | 1-5 | 1-5 | 1-2 | 1-5 |
| **Sleep** |  |  |  |  |
| Mean (SD) | 1.57 (0.959) | 1.46 (0.838) | 1.38 (0.652) | 1.32 (0.768) |
| Median | 1 | 1 | 1 | 1 |
| Range | 1-4 | 1-4 | 1-3 | 1-4 |
| **Daily routine** |  |  |  |  |
| Mean (SD) | 1.25 (0.645) | 1.36 (0.678) | 1.44 (0.613) | 1.18 (0.387) |
| Median | 1 | 1 | 1 | 1 |
| Range | 1-4 | 1-3 | 1-3 | 1-2 |
| **Joint activities** |  |  |  |  |
| Mean (SD) | 1.21 (0.499) | 1.54 (1.105) | 1.5 (1.052) | 1.47 (1.022) |
| Median | 1 | 1 | 1 | 1 |
| Range | 1-3 | 1-5 | 1-5 | 1-4 |
| N.B. Based on complete case analysis of the Child Health Utility-9 Dimension instrument data.  Scores rated as 1=least severe; 5=most severe.  Possible range for total scores: 9 (least severe across all 9 dimensions)-45 (most severe across all 9 dimensions). | | | | |

| **Supplemental Table 2. Multiple imputation outcomes totals and means (SD)** | | | | |
| --- | --- | --- | --- | --- |
| **Absenteeism and associated costs** | **Intervention (n=55)** | | **Control (n=59)** | |
|  | **Baseline** | **Follow up** | **Baseline** | **Follow up** |
| Healthcare Resource costs (£) |  |  |  |  |
| Total (95% CI) | 3051.83 (828.07; 5275.58) | 1145.09 (562.77; 1727.41) | 2315.32 (1324.16; 3306.49) | 1033.99 (510.86; 1557.11) |
| Mean (SD) | 55.49 (149.56) | 20.82 (39.16) | 39.24 (64.46) | 17.53 (34.02) |
| Child school absence |  |  |  |  |
| Total (95% CI) | 22.16 (8.17; 36.16) | 7.86 (3.09; 12.64) | 35.66 (19.60; 51.73) | 0.51  (-0.29; 1.33) |
| Mean (SD) | 0.40 (0.94) | 0.14 (0.32) | 0.60 (1.04) | 0.01 (0.05) |
| Parent work absenteeism |  |  |  |  |
| Total (95% CI) | 29.74 (6.22; 53.26) | 1.95  (-0.08; 3.98) | 9.59 (2.79; 16.39) | -0.19  (-0.65; 0.26)† |
| Mean (SD) | 0.54 (1.58) | 0.04 (0.14) | 0.16 (0.44) | -0.003 (0.03) † |
| Productivity costs (£) |  |  |  |  |
| Total (95% CI) | 3217.82 (673.32; 5762.31) | 211.21  (-8.71; 431.14) | 1037.90 (302.32; 1773.48) | -21.04  (-70.55; 28.47) † |
| Mean (SD) | -58.51 (171.35) | 3.84 (14.79) | 17.59 (47.84) | -0.36 (3.22) † |
| † Implausible figure; therefore, should be interpreted as zero. | | | | |

| **Supplemental Table 3. Multiple imputation of costs (£) and consequences related to intervention and control conditions** | | |
| --- | --- | --- |
| **Costs and consequences** | **Intervention (n=55)** | **Control (n=59)** |
| Child healthcare costs (£) |  |  |
| Mean difference (SD) between baseline and follow-up | -34.67 (148.86) | -21.72 (68.63) |
| 95% CI | -74.91; 5.57 | -39.60; -3.83 |
| Quality Adjusted Life Years |  |  |
| Mean difference (SD) between baseline and follow up | 0.22 (0.01) | 0.23 (0.01) |
| 95% CI | 0.22; 0.23 | 0.22; 0.23 |
| School absenteeism |  |  |
| Mean difference (SD) between baseline and follow-up | -0.26 (0.94) | -0.60 (1.04) |
| 95% CI | -0.51; -0.01 | -0.87; -0.32 |
| Workplace productivity due to child’s health (£) |  |  |
| Mean difference (SD) between baseline and follow-up | -54.67 (167.49) | -17.95 (47.59) |
| 95% CI | -99.95; -9.39 | -30.35; -5.55 |
